# Pulsed radiofrequency of the second cervical ganglion (C2) for the treatment of cervicogenic headache

**DOI:** 10.1007/s10194-011-0351-3

**Published:** 2011-05-25

**Authors:** Juan Zhang, Dong-sheng Shi, Rui Wang

**Affiliations:** Department of Anesthesiology, Traditional Medicine Hospital, Zhejiang University of Traditional Chinese Medicine, 54 Youdian Road, Hangzhou, 310006 People’s Republic of China

**Keywords:** Cervicogenic headache, Pulsed radiofrequency, C2 ganglion

## Abstract

In this case series report, two patients with cervicogenic headache were selected. After initial positive response to the greater occipital nerve block, pulse radiofrequency (PRF) was performed on the position of the second cervical ganglion (C2). Two patients reported 100% pain relief lasting for 6 months. The lateral puncture is safer and more comfortable than the posterior site. This case study demonstrates the effectiveness of PRF to treat cervicogenic headache originating from the C2 nerve. However, we need to further evaluate the results using more samples.

## Introduction

Headache is one of the common complaints for patients who seek treatment at the hospital. Acute and chronic headaches are a significant medical and socioeconomic problem. They are classified to a series of diseases, although the exact pathophysiology of headaches remains unknown. The term cervicogenic headache (CEH) was first introduced by Sjaastad et al. [1]. They also explained the syndrome and diagnostic criteria of CEH.

The classic CEH is characterized as recurrent, long-lasting, severe unilateral or bilateral headache originating from the neck. The common cause of CEH is the external pressure over the route of the greater and/or the lesser and or the third occipital nerve and the atlantoaxial subluxation. The injured the second cervical nerve (C2) and C1–C2 atlantoaxial joint or C2–C3 zygapophysial joint can also induce symptoms. The typical headache location is in the low occipital region and can radiate towards the periorbit and forehead. The neuroanatomical basis is the trigeminocervical nucleus in the spinal gray matter of the spinal cord at the C1–C3 level, where there is a convergence of the spinal cord neurons receiving both trigeminal and cervical input [2]. Therefore, symptom overlap between the trigeminal nerve and C1–C3 nerve is taken.

Anatomical studies have suggested that the C2 nerve may be more susceptible to injury than the other structures. Unlike the other cervical nerve, the C2 ganglion is not protected within a bony structure, but is covered by the atlantoepistrophic ligament. Postulated causes of compression and injury to this nerve include the following: (1) hypertrophy of the lamina or C1–C2 articulation, (2) osteoarthritis and spondylosis, (3) hypertrophy of the atlantoepistrophic ligament, and (4) the movement (rotary and extension) between the posterior arches and articular facets of atlas and axis [3–5]. The medial branch of the dorsal ramus of the C2 spinal nerve becomes the greater occipital nerve that classically innervates the occiput medially; therefore, the region of the greater occipital nerve dominates is the common region where the pain originates from the C2 nerve.

Because etiologies of headache are so diverse, it is critical for a correct diagnosis to be made prior to initiation of therapy. It is challenging to differentiate between CEH and other forms of headache because not only is there variability in headache presentation, but there is also considerable symptom overlap. CEH may be clinically diagnosed as migraines mistakenly. In addition to nausea and unilateral headache, neck pain is also a common symptom of migraines [6]. The International Headache Society and International Cervicogenic Headache Study Group have both developed different classification systems for the diagnosis of CEH. There are some slight differences between these classification systems. Except for the obvious disorder of the clinical, laboratory and/or imaging evidence accepted as a valid cause of headache, diagnostic blockade of its nerve is used to determinate the type of headache. The greater and even lesser occipital nerve injections have been recommended to diagnose and treat CEH.

Treatment modalities are wide and varied. Pharmaceutical options range from non-steroidal anti-inflammatory drugs to acetaminophen, but often yield inconsistent results and serious side effects. Nerve blocks may be utilized to relieve pain and elucidate the origin of pain; yet, they are only temporary measures. Epidural corticosteroid injections in CEH have also been endorsed [7]. However, the infection and symptoms associated with corticosteroid-related adverse effects make them unacceptable, the major method for the CEH population.

The conventional radiofrequency (CRF) as a neurodestructive treatment for pain uses a constant high frequency and high temperature. Unlike CRF, pulsed radiofrequency (PRF) delivers high intensity currents in pulses, making heat dissipate so that temperatures are not neurodestructive. It is neuromodulatory in nature and has not produced side effects, such as allodynia, hyperalgesia and dysesthesias. These advantages make it an excellent choice for the treatment of referred pain involving the C2 ganglion.

## Case report

Patients were referred to our pain clinic by the same doctor for treatment. The patients underwent a complete history and physical examination followed by diagnostic testing, such as X-ray and magnetic resonance image scan. These patients failed to take conservative therapy, including physical therapy, medications, and previous nerve blocks.

Initial diagnostic selective the greater occipital nerve blocks were performed with 1.5% lidocaine. Pain relief of 90% or more was accepted as a positive response to the nerve blocks lasting for at least 30 min. We performed the lateral pulsed radiofrequency application to the C2 ganglion.

**Fig. 1:**
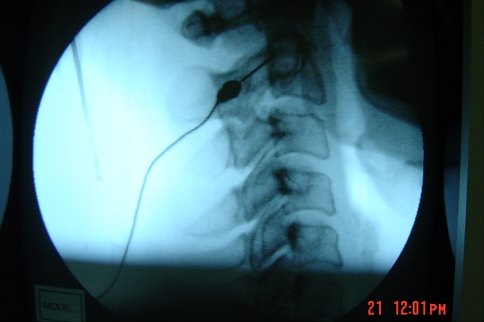
Lateral view of C2 with needle tip directed toward vertebra

The patient was placed in a supine position on the procedure table and the lateral neck area was prepped and draped with sterile towels. The skin was anesthetized with 1.5% lidocaine using a 25G needle with 0.5-mm diameter over the location of C-arm fluoroscopic guidance (Flexiview 8800). Sensory stimulation was then performed when the piercing needle reached the bone at 50 Hz to ascertain whether the pain is similar to the regular pain that was located in the dermatome of the occipital. Motor stimulation was then tested at 2 Hz to exclude the uncorrelated nerve. PRF was performed at 42° for 4 min as in Fig. 1. Two patients tolerated the procedure well and no significant complications occurred.

### Case 1

A 40-year-old woman suffered from left-sided posterior occipital pain. The pain was described as originated from the neck region and always extended to the area around her left eye for 5 years with the negative X-ray results. A decrease from 5 to 0 on 0–10 intensity scale lasted for a 6-month period of time.

### Case 2

A 66-year-old man presented with a history of left-sided posterior occipital pain with radiation in the periorbital, and parietal region sometimes for 1 year. A decrease from 4–0 on 0–10 intensity scale lasted for a 6-month period of time.

## Discussion

Cervicogenic headache is a known clinical syndrome with multiple causes, but the common treatment has most often consisted of blockade of the greater occipital nerve, the lesser occipital nerve, stellate ganglion block, and other various blocks [8]. Interruption of the cycle of pain and reflex muscle spasm are the most causes to relieve the symptoms; however, these effects have a short duration. Injection of depot methylprednisolone into the greater and lesser occipital nerve region produced complete headache relief for a period of 10–77 days [9].

Most uncommon treatments that involve radiofrequency cervical zygapophyseal joint neurotomy and C2 and or C3 ganglionectomies are destructive [10, 11]. Owing to the formation of neuromas, it is possible to cause subsequent hyperpathia and allodynia. All invariably cause sensory loss.

From the anatomy of the C2 nerve, we believed it to be the sole culprit for causing CEH. Deep cervical plexus block and C2 cervical nerve root block showed the efficiency to treat CEH [12, 13]; however, effective pain relief lasted for 3 months post-treatment, but by 6 months, the pain had returned to pre-treatment levels. The duration of pain relief is not satisfactory. Although C2 ganglionectomy is used as a method for long-term occipital neuralgia relief [11], as a neurodestructive treatment, it precipitates us to find a better therapy.

PRF treatment is supposed to be safer, and therefore, should be preferred to the treatment of CEH [14]. We used the PRF for the C2 ganglion and received pain relief for 6 months. Meanwhile, side effects have not been found in our therapy.

Serious complications can occur when cervical nerve root blocks are injected from posterior to frontal under the C-arm fluoroscopic guidance due to injection into the cervical subarachnoid space or into vessels in this region. Potential risks, include infection, stroke, paralysis and cerebrospinal fluid leak. We injected from the lateral toward the target of the C2 vertebra under the C-arm fluoroscopic guidance, which will result in fewer complications than the above method. Using the anterolateral approach for the C1–C2 joint injection, Halim et al. [15] also believed it would reduce the incidence of C2 nerve root injury, epidural injection, and vertebral artery puncture. The patients feel better in the supine position than in the prone position.

We treated our patients with PRF for C2 ganglion with successful results and without the potential neurodestructive side effects observed with CRF. However, additional studies should be undertaken to elucidate the nature of PRF so that we may more effectively apply this treatment to the patients.

PRF is a potential treatment option with good outcomes for patients suffering from CEH related to the C2 nerve. Although PRF provides shorter duration of pain relief than the CRF, it has fewer complications and is safer. The validity of this treatment needs to be proved further through more samples.

## References

[1] Antonaci F, Fredriksen TA, Sjaastad O (2001). Cervicogenic headache: clinical presentation, diagnostic criteria, and differential diagnosis. Curr Pain Headache Rep.

[2] Bogduk N (1992). The anatomical basis for cervicogenic headache. J Manipulative Physiol Ther.

[3] Stechison MT, Mullin BB (1994). Surgical treatment of greater occipital neuralgia: an appraisal of strategies. Acta Neurochi.

[4] Bogduk N (1999). C2 ganglion can be injured by compression between the posterior arch of the atlas and the lamina of C2. Spine.

[5] Poletti CE, Sweet WH (1990). Entrapment of the C2 root and ganglion by the atlanto-epistrophic ligament: clinical syndrome and surgical anatomy. Neurosurgery.

[6] Calhoun AH, Ford S, Millen C (2010). The prevalence of neck pain in migraine. Headache.

[7] He MW, Ni JX, Guo YN (2009). Continuous epidural block of the cervical vertebrae for cervicogenic headache. Chin Med J.

[8] Gawel MJ, Rothbart PJ (1992). Occipital nerve block in the management of headache and cervical pain. Cephalalgia.

[9] Anthony M (2000). Cervicogenic headache: prevalence and response to local steroid therapy. Clin Exp Rheumatol.

[10] Lee JB, Park JY, Park J (2007). Clinical efficacy of radiofrequency cervical zygapophyseal neurotomy in patients with chronic cervicogenic headache. J Korean Med Sci.

[11] Acar F, Miller J, Golshani KJ (2008). Pain relief after cervical ganglionectomy (C2 and C3) for the treatment of medically intractable occipital neuralgia. Stereotact Funct Neurosurg.

[12] Goldberg ME, Schwartzman RJ, Domsky R (2008). Deep cervical plexus block for the treatment of cervicogenic headache. Pain Physician.

[13] Dieterich M, Pöllmann W, Pfaffenrath V (1993). Cervicogenic headache: electronystagmography, perception of verticality and posturography in patients before and after C2-blockade. Cephalalgia.

[14] Van Boxem K, van Eerd M, Brinkhuizen T (2008). Radiofrequency and pulsed radiofrequency treatment of chronic pain syndromes: the available evidence. Pain Pract.

[15] Halim W, Chua NH, Vissers KC (2010). Long-term pain relief in patients with cervicogenic headaches after pulsed radiofrequency application into the lateral atlantoaxial (C1–2) joint using an anterolateral approach. Pain Pract.

